# Fluorescent Protein Approaches in Alpha Herpesvirus Research

**DOI:** 10.3390/v7112915

**Published:** 2015-11-19

**Authors:** Ian B. Hogue, Jens B. Bosse, Esteban A. Engel, Julian Scherer, Jiun-Ruey Hu, Tony del Rio, Lynn W. Enquist

**Affiliations:** Department of Molecular Biology & Princeton Neuroscience Institute, Princeton University, Princeton, NJ 08544, USA; ihogue@princeton.edu (I.B.H.); jens.bosse@hpi.uni-hamburg.de (J.B.B.); eengel@princeton.edu (E.A.E.); schererj@princeton.edu (J.S.); rueyhu@alumni.princeton.edu (J.-R.H.); tdelrio@bostonbiomedical.com (T.d.R.)

**Keywords:** alpha herpesvirus, herpes simplex virus, HSV, pseudorabies virus, fluorescent protein, GFP, fluorescence microscopy, capsid

## Abstract

In the nearly two decades since the popularization of green fluorescent protein (GFP), fluorescent protein-based methodologies have revolutionized molecular and cell biology, allowing us to literally see biological processes as never before. Naturally, this revolution has extended to virology in general, and to the study of alpha herpesviruses in particular. In this review, we provide a compendium of reported fluorescent protein fusions to herpes simplex virus 1 (HSV-1) and pseudorabies virus (PRV) structural proteins, discuss the underappreciated challenges of fluorescent protein-based approaches in the context of a replicating virus, and describe general strategies and best practices for creating new fluorescent fusions. We compare fluorescent protein methods to alternative approaches, and review two instructive examples of the caveats associated with fluorescent protein fusions, including describing several improved fluorescent capsid fusions in PRV. Finally, we present our future perspectives on the types of powerful experiments these tools now offer.

## 1. Introduction

In the nearly two decades since the popularization of green fluorescent protein (GFP), fluorescent protein (FP)-based methodologies have revolutionized molecular and cell biology, allowing us to literally see biological processes as never before. Naturally, this revolution has extended to virology in general, and to the study of alpha herpesviruses in particular. In this review, we provide a compendium of reported FP fusions to herpes simplex virus 1 (HSV-1) and pseudorabies virus (PRV) structural proteins, discuss the particular challenges of FP-based approaches in the context of a replicating virus, describe general strategies and best practices for creating FP fusions, review two instructive examples of the caveats associated with FP fusions, and present our future perspectives on the types of powerful experiments these tools now offer.

### 1.1. A Brief History of Fluorescent Proteins

GFP was first discovered in 1961 from *Aequorea victoria* jellyfish [1]. The GFP gene was cloned in 1992 [2], demonstrated to function as a transgene in other organisms in 1994 [3,4], and by 1995, many groups reported functional fusions between GFP and a variety of cellular proteins [5]. An enhanced GFP (EGFP) was described in 1996, widely disseminated, and remains the most commonly used “workhorse” FP today. EGFP contains mutations that increase expression in higher eukaryotes, improve protein folding, accelerate chromophore maturation, and shift its excitation maximum from 395 nm (ultraviolet) to 488 nm (blue-green). Variants were also engineered creating blue, cyan, and yellow FPs. Subsequently, the mutation A206K was identified, which suppresses the weak dimerization of EGFP variants [6]. Monomeric versions containing this mutation are frequently identified by a lowercase “m” prefix to the FP name (e.g., mEGFP). Many of the best FPs available today are only a few amino acids different from the parental EGFP.

The first orange-red fluorescent protein was not identified until 1999 [7]. DsRed, discovered in *Discosoma* sp. coral, was initially less useful than EGFP due to obligate tetramerization, aggregation, and very slow chromophore maturation. However, it did prove a fruitful precursor for the directed evolution of new FPs. A monomeric derivative, mRFP1 [8], was described in 2002 and became very popular. Further optimization has produced a variety of monomeric yellow, orange, red, and far-red FPs, many named for colorful fruits [9]. For example, mCherry [9] is currently the most popular red FP.

**Figure 1:**
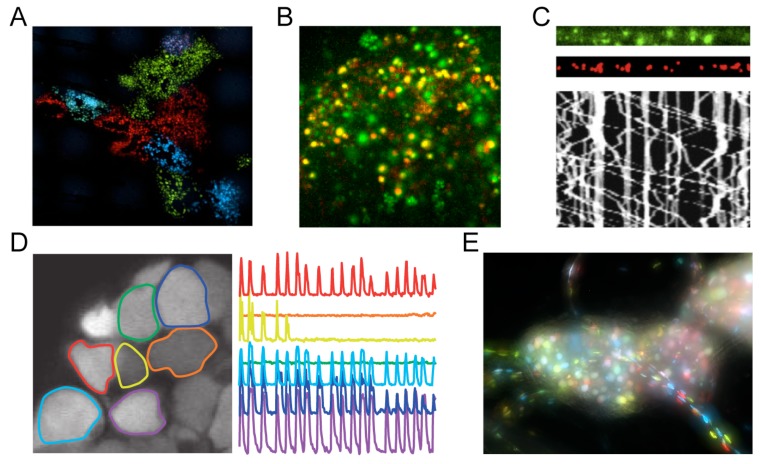
Examples of alpha herpesvirus research using fluorescent proteins and biosensors. (**A**) Three pseudorabies virus (PRV) strains expressing mCherry (red), enhanced yellow fluorescent protein (EYFP; depicted in green), or mCerulean (cyan) segregate to produce single-color plaques after spread from neurons [35]; (**B**) PRV expressing pHluorin, a pH sensitive FP biosensor (green), and mRFP1 fused to viral capsid protein VP26 (red), reveals the exocytosis of single virus particles [36]; (**C**) PRV expressing mRFP1 fused to viral capsid protein VP26 (red) co-transport with mCitrine-tagged kinesin-3 microtubule motors (green) in neuronal axons. Kymograph reveals movement of individual virus particles over time [37]; (**D**) PRV expressing GCaMP3, a Ca^2+^ biosensor, reveals synchronized firing of infected neurons *in vivo* [31]; (**E**) An explanted peripheral nervous system ganglion infected with a mixture of three PRV strains expressing mCherry (red), EYFP (green), or mCerulean (cyan) [38]. All images are reproduced with permission of original authors.

In addition to these two original families, FPs have also been developed from many other marine organisms. Some offer little advantage over more widely used and well-validated FPs, but a few do outperform EGFP and DsRed derivatives, as detailed below. Efforts are still underway to identify better FPs, and improved variants are reported every year. However, aside from improving general-purpose FPs, a major focus is now on the creation of specialized FP variants. Specialized FP variants include photo-activatable/switchable FPs [10,11], and “biosensors” designed to measure their local biochemical environment [12,13]. Examples of Ca^2+^ and pH biosensors are given in Section 1.2, Section 3.1.3, and Figure 1. Photo-activatable/switchable FPs are valuable for both ensemble studies (e.g., pulse-chase experiments using live-cell fluorescence microscopy), as well as single-molecule approaches due to the ability to stochastically switch single FP molecules (e.g., PALM super-resolution microscopy). Current photo-activatable/switchable FPs are more fully reviewed elsewhere [14,15].

### 1.2. Alpha Herpesviruses Strains Expressing Fluorescent Proteins and Biosensors

The first recombinant alpha herpesvirus expressing a FP was PRV [16], followed shortly by others, including HSV-1 [17], varicella-zoster virus (VZV) [18], and simian varicella virus [19]. Alpha herpesvirus strains expressing FPs are useful as cloning vectors for subsequent genetic manipulations of the virus, and facilitate basic virological research by allowing quick and easy visualization of live infected cells and tissues without the need to destructively fix and stain samples (e.g., Figure 1A).

In addition to FPs, alpha herpesviruses can also be engineered to express a variety of other transgenes to probe cellular and virological processes. In our experience, FP fusions to host proteins are not typically expressed well from the viral genome [20]. However, it was recently reported that synthetic introns and optimizing codon usage to match that of the GC-rich viral genome can enhance transgene expression in PRV [21].

In particular, PRV and HSV-1 strains expressing FPs have proven to be especially powerful and versatile tools in neuroanatomical research [22]. Alpha herpesviruses replicate and spread along chains of synaptically-connected neurons, and are among the very few viruses capable of efficiently spreading in both anterograde and retrograde directions. Unidirectional and non-replicating tracing strains also exist. For example, PRV Bartha and its FP-expressing derivatives spread only retrograde, the HSV-1 strain H129 is biased towards anterograde spread *in vivo* [23], and strains have been described that efficiently transduce neurons but do not replicate and spread [24]. See reference [22] for a list of available PRV-based neurotracing strains. PRV and HSV-1 strains have been engineered to conditionally replicate and express a fluorescent protein in subpopulations of neurons (e.g., Bartha2001 [25], and H129∆TK-TT [26]), or combinatorially express one of several different FPs (*i.e*., the “Brainbow” cassette [27]) under the control of Cre recombinase (see Section 2.3). These highly-specialized neural circuit tracing strains allow the kind of complex multifactorial experiments necessary to finely map neural circuits [25,28,29]. In addition to standard FPs, PRV strains have also been created expressing GCaMP Ca^2+^ biosensors, allowing researchers to simultaneously visualize neural connectivity and activity [30,31] (e.g., Figure 1D).

Originally, the fluorescent transgenes were inserted by replacing or disrupting non-essential viral genes, such as thymidine kinase, or glycoproteins gJ and gK in HSV-1 [17,32], and gG in PRV [16]. However, constructs have also been described in which the FP gene is inserted into non-coding intergenic regions. Insertions between UL3-UL4, UL26-UL27, UL50-UL51, and US1-US2 are all reported to be well tolerated in HSV-1 [33,34]. In each case, these intergenic regions exist between two converging transcriptional units (*i.e*., the flanking viral genes are oriented “tail-to-tail”), reducing the chances that a transgene insertion will produce polar effects on the surrounding genes.

### 1.3. Fluorescent Protein Fusions to Viral Proteins in HSV-1 and PRV

The alpha herpesviruses have been studied for decades using classical virological, biochemical, genetics, and electron microscopy approaches [39]. These types of studies have yielded a broad understanding of virion composition and the major steps of the virus replication cycle. However, these classical methods typically lack the spatiotemporal resolution to fully understand the kinetics and dynamics of viral processes. FP-based techniques fill this gap, allowing dynamic viral processes to be imaged over a time course in live infected cells (e.g., Figure 1B,C).

**Figure 2:**
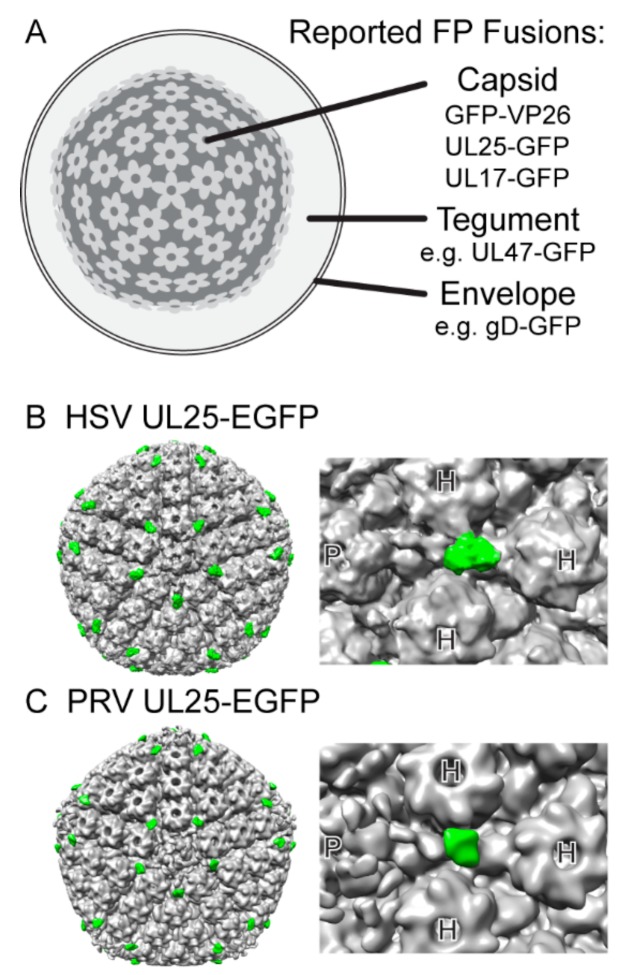
Structure of the Alpha Herpesvirus particle. (**A**) Virions are composed of an icosahedral capsid containing the viral genome, a complex and pleomorphic tegument, and pleomorphic envelope. See Table 1 for a complete list and references of viral structural proteins reported to tolerate fluorescent protein fusion. (**B,C**) Enhanced green fluorescent protein (EGFP) fusions to viral capsid protein UL25 can be detected by cryo electron microscopy in herpes simplex virus 1 (HSV-1) and pseudorabies virus (PRV). Five copies of UL25 are bound to each penton. Due to the elongated conformation of UL25 [98], the EGFP moiety (green) appears distal to the penton (P), between hexons (H). The EGFP density thus serves as a convenient mass tag to aid in determining the location of particular viral proteins on the capsid. (**B**) A 13.7 Å icosahedral reconstruction of an HSV-1 nuclear C-capsid, rendered from EMDB-1904, originally published by Conway *et al.* [99]. (**C**) A 23.2 Å icosahedral reconstruction of a PRV nuclear C-capsid, rendered from EMDB-5657, originally published by Homa *et al.* [100].

The first reported viral protein-FP fusion among the alpha herpesviruses was EGFP fused to the HSV-1 tegument protein VP22, described by Elliott and O’Hare in 1997 [40]. This was followed shortly by an EGFP fusion to the small capsid protein, VP26, described by Desai and Person in 1998 [41]. In this case, the authors monitored the accumulation of capsid proteins in the nucleoplasm and cytoplasm during infection, but were not yet able to resolve individual capsids. Since then, there have been many functional FP fusions reported, including fusions to envelope, tegument, and capsid structural proteins (see Figure 2 and Table 1), as well as many non-structural proteins.

**Table 1:** Fluorescent protein fusions to structural proteins in HSV-1 and PRV.

Gene/Protein Name	Description/Function	Structural Role	Fusion Location	References	
				HSV-1	PRV
**Capsid Proteins**					
UL17	capsid vertex specific component	capsid	*C*-terminal	[42]	
UL25	capsid vertex specific component	capsid	internal	[43]	[44]
UL35 VP26	small capsid protein	capsid	*N*-terminal	[41,45]	[46]
			*C*-terminal		[47]
**Tegument Proteins**					
UL13	protein kinase	tegument	*N*-terminal		[48]
			*C*-terminal		[48]
UL16	interacts with UL21	tegument	*C*-terminal	[49]	[50]
UL21	interacts with UL16		*N*-terminal	[51]	
			*C*-terminal	[52]^b^	
UL31	nuclear egress	tegument ^a^	*N*-terminal	[53,54]	[55]
UL36 VP1/2	large tegument protein	tegument	*N*-terminal		[56]
			*C*-terminal	[57]	[44]
UL37	interacts with UL36	tegument	*N*-terminal	[57]	
			*C*-terminal	[58]	[56]
UL41 VHS	RNAase	tegument	*N*-terminal	[59]	
UL46 VP11/12	most abundant tegument protein	tegument	*N*-terminal	[57]	
			*C*-terminal	[60]	[50]
UL47 VP13/14		tegument	*N*-terminal	[61]	[56]
UL48 VP16	transactivation of viral gene expression	tegument	*N*-terminal	[62]	
			*C*-terminal	[57,62]	[56]
UL49 VP22	secondary envelopment	tegument	*N*-terminal	[57,63]	[56,64]
			*C*-terminal	[40,63]	[64]
US1 ICP22	regulation of viral gene expression	tegument	*C*-terminal	[65]	
US3	protein kinase	tegument	*N*-terminal	[66]	
			*C*-terminal	[67]^b^	[48,68]
US10	unknown	tegument	*C*-terminal		[69]^c^
ICP34.5		tegument	*N*-terminal	[70]	*
ICP0 (EP0)	transactivation of viral gene expression, E3 ubiquitin ligase	tegument	*N*-terminal	[71]	
			*C*-terminal	[72]	[73]
			internal	[74]	
ICP4 (IE180)	transactivation of viral gene expression	tegument	*N*-terminal	[75]	
			*C*-terminal	[76]	[77]
**Envelope Proteins**					
UL10 gM	glycoprotein M	envelope	*N*-terminal, intravirion	[78]	[78]
			
			*C*-terminal, intravirion		[36,79]
			internal, extravirion		[36]
UL11	lipid-anchored membrane protein	envelope	*C*-terminal	[49]	[49]
UL20	multipass transmembrane protein	envelope		[80]	
UL22 gH	glycoprotein H, membrane fusion	envelope	*N*-terminal, extravirion	[81]	
UL27 gB	glycoprotein B, receptor binding, membrane fusion	envelope	*N*-terminal, extravirion	[82,83]	
			*C*-terminal, intravirion	[84]	
UL34	transmembrane protein, nuclear egress	envelope ^a^	*C*-terminal		[85]^d^
UL43	multipass transmembrane protein	envelope	*C*-terminal, intravirion		[86]^d^
UL44 gC	glycoprotein C, receptor binding	envelope	*C*-terminal, intravirion		[87]
UL49A gN	glycoprotein N	envelope	*C*-terminal, intravirion	[88]	
UL51	lipid-anchored membrane protein	envelope	*C*-terminal	[89]	
UL53 gK	glycoprotein K	envelope	*C*-terminal, extravirion		[90]^d^
US2	lipid-anchored membrane protein	envelope	*N*-terminal	[91]	
US6 gD	glycoprotein D, receptor binding, membrane fusion	envelope	*C*-terminal, intravirion	[92]	[93]
US7 gI	glycoprotein I, anterograde axonal transport	envelope	*N*-terminal, extravirion		[94]
US8 gE	glycoprotein E, anterograde axonal transport	envelope	*N*-terminal, extravirion		[94]
			*C*-terminal, intravirion		[95]
US9	transmembrane protein, anterograde axonal transport	envelope	*N*-terminal, intravirion	[37]	[79]

Notes: Viral proteins incorporated into virus particles determined by mass spec proteomics approaches [96,97]. ^a^ present in primary enveloped particles, but not mature enveloped particles; ^b^ fusion reported in HSV-2; ^c^ fusion reported in Marek's disease virus; ^d^ fusion reported in equine herpesvirus 1; * PRV does not encode an ortholog of this gene/protein.

## 2. Unique Challenges of Fluorescent Protein-based Approaches in Viruses

### 2.1. Structure of Virion

The mature icosahedral herpesvirus capsid is composed of the major capsid protein (VP5), triplex proteins (VP19C and VP23), and the portal protein (UL6). Due to strict structural and stoichiometry requirements, FP fusion may not be well tolerated. Our attempt at creating FP fusion to VP5 in PRV was partially successful: a VP5-mEGFP fusion is tolerated only when co-expressed with wild-type, unfused VP5 [101]. The small capsid protein (VP26) and capsid vertex-specific components (UL25 and UL17) are peripherally associated with the capsid and do tolerate FP fusions. Tagging VP26 is currently the most common method to visualize alpha herpesvirus capsids in fluorescence microscopy. FP-tagged VP26 incorporates hundreds of copies, producing a brightly fluorescent capsid; however incorporation is somewhat variable and less than the 900 molecules predicted to be on a wild-type capsid [44]. In addition, VP26 fusions cause a 0.5-1 log defect in virus replication (see Figure 5), and reduce neurovirulence *in vivo* [102]. In contrast, FP-tagged UL25 is incorporated with a more strictly defined 60 copies per capsid (see Figure 2), and does not cause detectable replication or pathogenicity defects *in vitro* or *in vivo* [44,50,99,100]. UL17 is also reported to tolerate FP fusion, but appears to incorporate about 30% fewer copies than UL25 [42]. Finally, there is an unpublished report of an FP fusion to the scaffold protease (VP24), which remains inside capsids after assembly, which may facilitate visualization of procapsids prior to VP26 or UL25 incorporation [103].

In contrast to the capsid, most of the virion tegument and envelope are pleomorphic. While this lack of strict structure and stoichiometry may allow the virus to better tolerate FP fusions, variable incorporation of these proteins may produce misleading experimental results or unappreciated mutant phenotypes (see Section 4 below for illustrative examples). See Figure 2 and Table 1 for a list of reported FP fusions to HSV-1 and PRV structural proteins.

### 2.2. Genome Constraints

Even if viral proteins and protein complexes can tolerate FP fusion, the genome structure may not. Although not as compact as RNA viruses, alpha herpesvirus genomes contain many overlapping genes. Promoter and other *cis*-acting elements often overlap transcribed and coding regions, and there are many co-terminal transcripts in which multiple genes share the same polyadenylation signal. For example, the gG (US4) locus is a common place to insert transgenes into the PRV genome. This locus is transcribed as two co-terminal transcripts, the first expressing the US3 protein kinase, and the second expressing gG. Transgenes inserted in place of gG are also transcribed in the 3’ UTR of the US3 transcript, which has been shown in some cases to reduce US3 protein expression [104]. Such potentially disruptive polar effects are difficult to predict, as viral promoters and other *cis*-acting elements are not well identified.

Another potential problem is that FP sequences themselves may contain cryptic *cis* elements. For example, the original *Aequorea* GFP sequence contained a cryptic intron that limited its expression in plants [105], and Katushka (the ancestor of mKate2 and others) contains a cryptic splice donor that can cause unexpected RNA splicing in mammalian cells [106].

However, inadvertent effects of transgene insertion are not always disadvantageous. As discussed more fully below, the original HSV-1 VP26 capsid fusion, reported by Desai *et al.* [41], contains an inadvertent deletion of an upstream promoter element. The resulting decrease in fusion protein expression is actually beneficial for viral replication [45].

### 2.3. Evolvable and Highly Recombinogenic Genomes

Just like cellular life, viruses are replicating genetic entities that are subject to the pressures of natural selection; in short, they are evolvable. The virus genome can acquire point mutations by the intrinsic error rate of its viral polymerase, environmental DNA damage, and interactions with the host DNA repair machinery or intrinsic immune defense mechanisms (e.g., APOBEC [107,108]). In addition, the alpha herpesvirus genomes contain many repetitive sequences and are highly recombinogenic. It has been suggested that this ability to expand and contract repeats by homologous recombination allows a virus population to more rapidly generate genetic diversity and more efficiently explore the fitness landscape, an idea referred to as the “genetic accordion” hypothesis [109]. With this relatively high recombination rate and very large population sizes, introducing a transgene that is disadvantageous in any way can rapidly select for reversions, deletions, and compensatory mutations.

**Figure 3:**
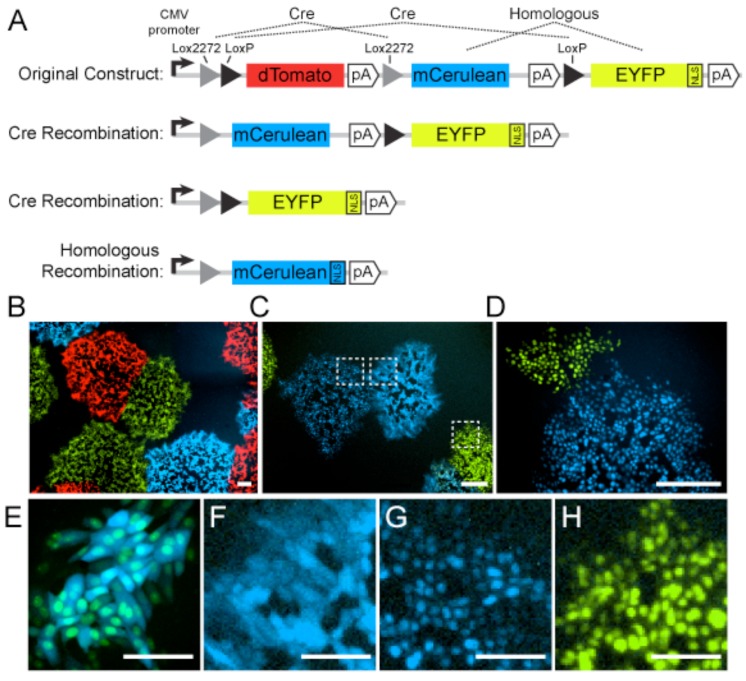
Recombination between homologous fluorescent proteins. (**A**) Herpes simplex virus 1 (HSV-1) 17syn+ strain containing the Brainbow 1.0L cassette inserted into the UL37/UL38 intergenic region. The original construct expresses dTomato (red). Upon co-expression with Cre recombinase, site-specific recombination at Lox sites produces progeny virus genomes that express either mCerulean (cyan) or enhanced yellow fluorescent protein (EYFP; depicted in green) fused to a nuclear localization signal (NLS). Due to the high sequence similarity between mCerulean and EYFP, non-Cre homologous recombination can transfer the NLS to mCerulean, producing an unexpected phenotype. (**B**) HSV-1 plaques expressing dTomato, mCerulean, or EYFP-NLS. (**C**) HSV-1 plaques expressing mCerulean, EYFP-NLS, or unexpectedly, mCerulean-NLS. Boxed regions are magnified in panels F-H. (**D**) HSV-1 plaques expressing EYFP-NLS, or unexpectedly, mCerulean-NLS. Scale bars in panels B-D are 250 µm. (**E**) Cells co-infected with HSV-1 expressing mCerulean and EYFP-NLS, illustrating the distinction between cytosolic and nuclear localization. (**F**) Cells infected with HSV-1 expressing cytosolic mCerulean, as expected. (**G**) Cells infected with HSV-1 unexpectedly expressing mCerulean-NLS. (**H**) Cells infected with HSV-1 expressing EYFP-NLS, as expected. Scale bars in panels E–H are 100 µm.

Inserting multiple homologous FPs can lead to frequent recombination and exchange of FP properties, a phenomenon previously described as “GFP walking” [110]. Given the highly recombinogenic nature of alpha herpesviruses, we previously predicted that this phenomenon would occur more frequently in alpha herpesvirus genomes [111]. Here we quantify this phenomenon by constructing an HSV-1 17syn+ mutant expressing the Brainbow 1.0L cassette [27], which contains multiple homologous FPs and other repeated sequences. HSV-1 17syn+ is a non-syncytial variant of Glasgow strain 17, and is commonly used as a laboratory wild-type HSV-1 strain [112]. In Vero cells, we measured the rate of intergenic non-Cre-mediated homologous recombination between EYFP and mCerulean, which share 97% nucleotide identity. To identify these recombinants, we inserted a nuclear localization sequence (NLS) at the C-terminus of EYFP. As expected, yellow fluorescence was observed only in the nuclei of infected cells after Cre-mediated recombination (Figure 3). However, due to non-Cre-mediated homologous recombination, the NLS expression pattern was unexpectedly detected in 12% of virus clones expressing mCerulean. In a total of 200 mCerulean-expressing viral plaques, 24 exhibited nuclear localization of mCerulean.

Similar unexpected rearrangements can happen when intramolecular tandem dimer FPs, like tdTomato [9], are used in alpha herpesvirus genomes. tdTomato may recombine to produce dTomato, which forms intermolecular dimers. If tdTomato is fused to a viral protein of interest, recombination may produce unexpected and undesired dimerization of that viral protein.

When several FPs are cloned into a viral genome, the chances of undesired recombination, rearrangements, or deletions can be minimized by carefully choosing FP with low sequence homology. Newer Brainbow 3.0 cassettes have partially solved some of the problems mentioned above. Instead of homologous *Aequorea*-derived FPs, the Brainbow 3.0 cassettes express FPs chosen to aggregate less *in vivo*, remain fluorescent after chemical fixation, and that are antigenically distinct. This latter property allows FPs to be distinguished by antibody staining when necessary, and also reduces the risk of intergenic homologous recombination when inserted into viral or cellular genomes [113].

## 3. How-To: General Strategies and Best Practices

### 3.1. Choice of Fluorescent Protein

The best general-purpose FPs are optimized to be bright, stable, and relatively insensitive to their surroundings. The Nikon Imaging Center at UCSF provides an excellent overview (nic.ucsf.edu/FPvisualization/). These FPs are most appropriate for the types of experiments where fluorescence intensity is assumed to be representative of the local concentration of the fusion protein of interest. In contrast, some FPs are optimized for specialized purposes, such as FP biosensors that are designed to be more sensitive to their surroundings. Moreover, there are often trade-offs in FP optimization: highly optimizing one property may come at the detriment of other properties. Therefore, the newest and most highly optimized FP variants may not be the best, depending on the particular experimental demands. In addition to general considerations, like excitation and emission spectra, brightness, and photostability, below we highlight several FP properties that we have found to be often overlooked, yet particularly important in the study of viruses.

#### 3.1.1. Fluorescent Protein Dimerization

With some viral proteins present at many hundreds of copies per virion, high local FP concentration can intensify problems associated with weak FP dimerization. For example, there are reported to be ~450 copies of GFP-tagged tegument protein VP13/14 (UL47) per virion [50]. If these molecules are evenly dispersed in the tegument volume, the local concentration of GFP would be approximately 0.1 mM. This high local concentration is near the dissociation constant of EGFP derivatives that lack the monomerizing A206K mutation (K_d_ ~0.1 mM). Thus, within the confines of the virion, some FP variants may function predominantly as a dimer. This weak dimerization affinity has indeed been reported to interfere with capsid assembly in HSV-1 [45], and we report a similar phenomenon in PRV, as discussed in Section 4.2.

#### 3.1.2. Fluorescent Protein Maturation Time

Within a newly translated FP molecule, critical residues must undergo an oxidation reaction to form the fluorophore, a rate-limiting step that is 1–2 orders of magnitude slower than protein folding. Maturation rates reported in the literature are measured by a variety of methods; as a result, rates are not always directly comparable, and popular FP variants have a range of reported values. Nevertheless, maturation half-times reported in Table 2 provide an estimate of whether a FP matures quickly. The blue, cyan, green, yellow, and far-red FPs, including most of the *Aequorea*-derived FPs, mature on the order of tens of minutes. The popular mRFP1 and mCherry also mature quickly, but other DsRed derivatives, like mOrange2, require many hours. While brighter and more photostable than mRFP1 and mCherry, the newer red FPs TagRFP-T and FusionRed are reported to mature somewhat more slowly (Table 2).

Compared to many other viruses, including most of the beta and gamma herpesviruses, the alpha herpesviruses have a relatively fast replication cycle. In HSV-1, immediate-early gene expression peaks at 2–4 h post infection (hpi), early gene expression and DNA replication is detectable as early as 3 hpi [114], and progeny virus appears by 6 hpi in single-step replication assays. PRV is even faster: using single-particle fluorescence microscopy techniques, we have observed the assembly of capsids in the nucleus as early as 3.25 hpi and the exocytosis of enveloped virions at the cell surface as early as 4.5 hpi [36,115]. With such a fast replication cycle, the time it takes for a FP to mature and become fluorescent becomes very important.

**Table 2:** Maturation time (t_1/2_) of selected fluorescent protein variants.

Fluorescent Protein	t_1/2_ (min)	References
Blue		
mTagBFP2	12	[116]
Cyan		
SCFP3A	82	[117]
mCerulean3, mTurquoise2	^a^	[118]
Green		
wild-type *Aequorea* GFP	37–83 ^b^	[119,120]
EGFP, mEGFP	10–65 ^b^	[120,121]
Emerald	8	[120]
mNeonGreen	<10	[122]
TagGFP2	18	[123]
Yellow		
Venus, mVenus	28–72 ^b^	[117,120]
SYFP2	55	[117]
Orange		
mKO2	108	[124]
mOrange2	270	[125]
Red		
DsRed	~600	[9]
mRFP1	<60	[9,125]
mCherry	15–40 ^b^	[9,125,126]
mStrawberry	50	
TagRFP-T	100	[125]
FusionRed	130	[127]
Far-Red		
mKate2	<20	[126]
mNeptune	35	[128]

^a^ similar kinetics as SCFP3A *in vivo;* ^b^ different values are reported in the literature based on different methods.

**Figure 4:**
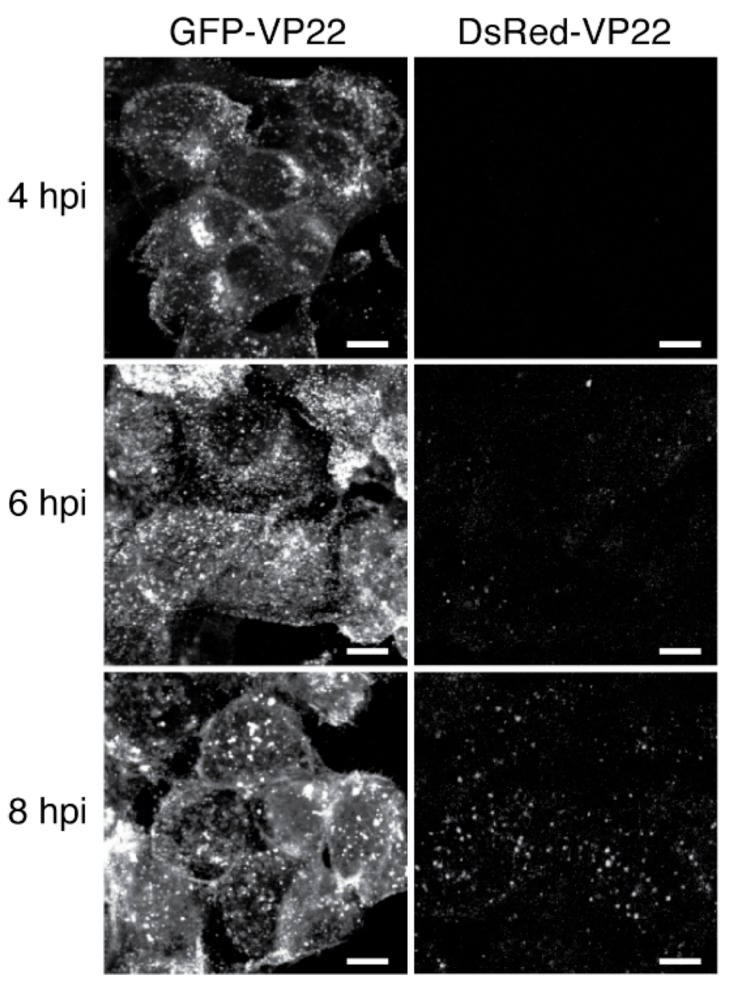
Slow-maturing DsRed does not accurately reflect viral protein expression. PK15 cells were infected with pseudorabies virus (PRV) strains expressing EGFP-tagged VP22 (left column), or DsRed-tagged VP22 (right column), and imaged at 4, 6, and 8 hpi. Because of its fast fluorophore maturation (EGFP t_1/2_ = tens of minutes), EGFP-tagged VP22 can be readily detected by confocal microscopy at 4 h post-infection (hpi). DsRed-tagged VP22 is not efficiently detected throughout the course of infection likely due to slow fluorophore maturation (DsRed t_1/2_ = ~10 h). Scale bars represent 10 μm.

As an extreme example, the maturation half time of the ancestral DsRed FP is around 10 h, by which time the alpha herpesvirus replication cycle is nearly complete. In Figure 4, PK15 cells were infected with PRV strains expressing EGFP-tagged VP22 or DsRed-tagged VP22. The EGFP-tagged version produced nuclear and cytosolic puncta by 4 hpi. In contrast, the slow maturation of DsRed is not well matched to time-course of virus infection: DsRed-VP22 is undetectable at 4 hpi, and only beginning to reveal intracellular puncta at 8 hpi, near the end of the replication cycle (Figure 4). Modern FP variants that mature in minutes are better suited to study early events in viral replication than those requiring hours.

#### 3.1.3. Fluorescent Protein pH Sensitivity

Most intracellular membranes are acidified by the action of vacuolar ATPases, which pump protons into the lumen of these organelles. Cargo of the secretory pathway experiences a gradient of pH, from near neutral in the ER, moderately acidified in the Golgi, to most acidic in post-Golgi secretory vesicles. Endocytic cargo is also exposed to a pH gradient, from moderately acidic early and recycling endosomes maturing into more acidic late endosomes, to most acidic in the lysosomes (Table 3).

**Table 3:** Typical pH of intracellular compartments.

Compartment	pH	References
Cytoplasm	7.2–7.4	[129,130]
Nucleus	7.4–7.8	[131,132]
Secretory Pathway		
Endoplasmic Reticulum	7.2	[129]
*cis*-Golgi	6.7	[129]
*trans*-Golgi	6.0	[129]
Secretory Vesicles	5.2–5.7	[129]
Endocytic Pathway		
Early and Recycling Endosomes	6.3–6.5	[129]
Late Endosomes	6.0	[129]
Lysosomes	5.5	[129]

Herpesviruses can enter cells via endocytosis in some cell types, viral membrane proteins are synthesized in the secretory pathway, and secondary envelopment and egress of virus particles occurs at trans-Golgi, endosomal, and/or secretory vesicle membranes. Thus, all viral structural proteins pass though acidic compartments at some point in the virus replication cycle. Therefore, when creating new viral FP fusions, it is important to consider how FPs will behave in these acidic compartments.

In general, the current generations of blue, cyan, and red FPs are relatively pH insensitive (Table 4). With pKa values less than 4.5, these FPs will retain most of their fluorescence in even the most acidic intracellular compartments. To image viral proteins and particles in acidic organelles, these FPs are the best choice.

**Table 4:** pH sensitivity (pKa) of selected fluorescent protein variants.

Fluorescent Protein	pKa	Notes	Reference
Blue			
mTagBFP2	2.7		[116]
Cyan		as a class, cyan FPs are relatively pH insensitive	
ECFP, mCerulean	4.7		[133]
mTFP1	4.3		[134]
mCerulean3	3.2		[135]
mTurquoise2	3.1		[118]
Green		as a class, green FPs are moderately pH sensitive	
mEGFP, mEmerald	6.0		[136]
mNeonGreen	5.7		[122]
mTagGFP2	5.0		[123]
T-Sapphire	4.9	a UV-excited green FP	
superecliptic pHluorin	7.2	purposely optimized to be very pH sensitive	[137]
Yellow		as a class, modern yellow FPs are moderately pH sensitive	
EYFP	6.9	very pH sensitive, newer variants are somewhat improved	
mVenus, SYFP2	6.0		[117,138]
mCitrine	5.7		[136]
Orange			
mKO2	5.0–5.5		[125]
mOrange, mOrange2	6.5–7.5	very pH sensitive	[125]
Red		as a class, red FPs are relatively pH insensitive	
mRFP1, mCherry, mStrawberry	≤4.5		[9]
TagRFP-T	4.0		[125]
FusionRed	4.6		[127]
Far-Red			
mKate2	5.4		[126]
mNeptune	5.8		[128]

Current green, yellow, and far-red FPs are moderately pH sensitive. The most popular green FP, EGFP, has a pKa of 6.0, meaning it will lose around half of its fluorescence in moderately acidic compartments like the trans-Golgi network. Historically, most herpesvirus structural proteins have been tagged with EGFP, and the potential for experimental artifacts due to the pH sensitivity of these fusions is underappreciated in the literature. With slightly lower pKa, T-Sapphire, a UV-excited GFP derivative, mTagGFP2, derived from another *Aequorea* species, and mNeonGreen, derived from another marine animal, may slightly outperform EGFP under acidic conditions. We have successfully used mNeonGreen as a VP26 (UL35) capsid tag in PRV (see Section 4.2).

Other FPs are highly pH sensitive, limiting their use in the study of virus proteins or particles in acidic compartments. EYFP, an older-generation yellow FP, is especially sensitive to pH. Newer yellow derivatives (e.g., mVenus and mCitrine) are improved, although still moderately pH sensitive. Many of the less-common mFruit derivatives (e.g., mBanana, mOrange2, mTangerine, and mApple) are also very pH sensitive (pKa 6.5–6.7). Circularly permuted GFP derivatives, which are used to create FP biosensors, such as the GCaMP Ca^2+^ sensors, are also especially pH sensitive. The pH sensitivity of such FP biosensors has indeed led to experimental artifacts [139]. However, the pH sensitivity of FPs can also be exploited as an experimental tool. For example, EYFP has been used as a pH sensor to study retrovirus fusion and entry [140], and several GFP variants, named pHluorins, have been purposely optimized as pH sensors. With a pKa of 7.2, superecliptic pHluorin is strongly quenched in the secretory pathway, but dequenched upon exocytosis, allowing researchers to image individual exocytosis events [137,141], including the egress of PRV particles from infected cells [36] (Figure 1B).

### 3.2. Choice of Fluorescent Protein Insertion Site and Linker

Many of the published FP fusions in the alpha herpesviruses were created using laborious restriction digestion and ligation-based methods. This approach frequently produces arbitrary junctions between FP and viral sequences determined by the presence of convenient restriction sites. As a result, many existing FP fusions contain insertions of restriction sites or plasmid-derived sequences, and deletions or duplications in flanking viral genomic sequences. Nevertheless, many viral FP fusions created this way worked very well, sometimes better than subsequent rationally-designed fusions. Today, improved methods, such as traceless bacterial artificial chromosome (BAC) recombination [142], and the ability to synthesize whole synthetic genes to order, provide greater flexibility to rationally design FP fusion proteins. Yet, even when detailed FP and viral protein structures are known, we are unable to accurately predict structure and function, so creating a fully functional FP fusion remains largely a process of trial and error. Nonetheless, several general considerations may improve the probability of creating a functional fusion.

Foremost, previous reports of functional FP fusions can be a valuable guide. Since approximately 40 core genes are conserved throughout the herpesviruses, and even more are conserved within the alpha herpesviruses, a similar FP fusion may already exist in a related herpesvirus. Table 1 may facilitate this search.

To design a novel fusion, it is worth considering any known structural or functional motifs to avoid interfering with protein function. The UniProt Knowledgebase (www.uniprot.org) is a useful repository of this sort of information for both viral and host proteins. If any structure is known, inserting a FP into unstructured termini or loops may reduce the chances of the FP interfering with protein structure. When no empirical data is available, structure prediction software can estimate some features with varying degrees of certainty. For example, prediction of transmembrane domains is relatively robust. Unstructured hinges or loops, possibly providing good FP insertion sites, may flank such strongly predicted structural motifs. In addition, regions with an abundance of hydrophilic residues are generally more likely to be exposed to the solvent on the surface of proteins, and thus may be better insertion sites than buried hydrophobic regions.

Finally, consider whether to insert a linker peptide between the FP and protein of interest. It is not always necessary to add linkers, as most FPs contain unstructured residues on their N- and C-termini that may provide adequate flexibility and spacing. However, when structural or steric conflicts do occur, adding additional flexible linker residues between the FP and gene of interest may help. Linkers containing residues that are small (glycine, alanine) or polar (serine, threonine, asparagine) may favor the formation of unstructured flexible loops, and proline residues disfavor the formation or extension of alpha helices. 

### 3.3. Choice of Excitation Light Sources

Excitation sources for fluorescence microscopy have changed dramatically in recent years, transitioning from arc lamps and gas lasers to light emitting diodes (LEDs), laser diodes, and diode pumped solid state (DPSS) lasers. These new sources have major advantages in efficiency, total power, and direct modulation; however, they also present unique challenges to integrate their use with existing FPs.

To excite green FPs, powerful DPSS lasers can replace argon gas lasers as a source for 488 nm laser illumination; however, 488 nm DPSS lasers remain costly. 473 nm DPSS lasers are an inexpensive alternative, resulting in only slightly less excitation efficiency of EGFP and similar derivatives. There are currently few LEDs available in the 490 nm range, and their efficiency is inferior to the much more common 470 nm LEDs. As most LEDs have relatively broad emission spectra, 470 nm LEDs typically work well with commonly-used multiband excitation filters, and are a good alternative to arc lamps.

The demise of argon gas lasers presents a challenge for those using cyan and yellow FPs. Argon gas lasers produce 454.6 nm and 514.5 nm laser lines, which efficiently excite cyan and yellow FPs, respectively. Now, microscopes typically use 405 nm laser diodes and 488 nm DPSS lasers, which are both suboptimal for these FPs. This is unfortunate because some of the newest FPs in this range, like mTurquoise2 [118], are among the brightest and most monomeric FPs available. However, recent advances have made laser diodes in the 445 and 515 nm range very cost-effective. Still, these are rarely integrated into commercial microscopes. On custom built systems, these laser lines can be added relatively easily and inexpensively. Another big advantage of these diodes is that they can be modulated directly, omitting the need for costly acousto-optical tunable filters (AOTF). High power LEDs centered around 450 nm and 505 nm are readily available, and are a good alternative to arc lamps.

For red FPs, there are currently no widely-available laser diodes in the 560–590 nm range. Instead, 561 nm DPSS lasers are typically used on commercial microscopes. While this wavelength is optimal for common chemical fluorophores, it is suboptimal for mCherry and other red FPs. However, 589 and 593 nm DPSS lasers do exist, and could be a better option for imaging red FPs. Very few LEDs exist in the 540–580 nm range, and most are phosphor-converted LEDs that produce broad spectra with low peak power. Amber LEDs centered around 590 nm are available; however, they are relatively weak, and older arc lamps generally out-perform LEDs when imaging red FPs. Future developments in diode technology will hopefully close this gap in this spectral region.

## 4. Case Studies: Discussion of Particular Fluorescent Protein Fusions

### 4.1. PRV Membrane Protein US9

Our laboratory has long been interested in the axonal transport and trans-neuronal spread of alpha herpesviruses. While many viruses can invade the nervous system, for most it is a non-productive dead-end pathway. The alpha herpesviruses are among the very few that have evolved to efficiently enter and productively exit the nervous system of their natural hosts. Accordingly, while many viruses are capable of entering at nerve termini and undergoing retrograde axonal transport post-entry, the alpha herpesviruses are among the very few capable of efficient anterograde axonal transport of replicated progeny [143].

The attenuated vaccine strain, PRV Bartha, provided our first toehold to begin to understand the molecular mechanisms of alpha herpesvirus anterograde axonal transport and spread. PRV Bartha contains a large, 3 kb deletion in the unique short (US) genomic region, disrupting viral proteins gI (US7), gE (US8), US9, and US2. Of these disrupted genes, US9 is essential for anterograde axonal transport of progeny virions in infected neurons [144,145], and similar phenotypes exist in HSV-1. US9 is a small, 98 amino acid, viral membrane protein. The cytosolic N-terminal domain of US9 contains many functional motifs, including phosphorylation sites [146], endocytic motifs [147], and it is thought to recruit the kinesin microtubule motor KIF1A (kinesin-3) [37] in concert with gE/gI [95]. The transmembrane domain is located near the C-terminus, producing a very small, 3 amino acid extracellular domain with no known functional motifs.

Originally, our laboratory fused EGFP to the short C-terminal domain to avoid interfering with the functional motifs of the cytosolic domain [148]. This US9-EGFP fusion protein was efficiently incorporated into virus particles, and its intracellular transport and localization was indistinguishable from that of wild-type US9 [147,148]. Together, these observations suggested that the US9-EGFP fusion was indeed functional. However, an important caveat is that US9-null viruses do not have a detectable phenotype in non-neuronal cells. It was only once we constructed a recombinant virus expressing this US9-EGFP fusion and assessed its neuronal spread *in vitro* and *in vivo* did its mutant phenotype become apparent: the US9-EGFP fusion protein is not functional in anterograde axonal transport in neurons [149]. Thus, our experience with EGFP-tagged US9 serves as an instructive example of the major pitfalls associated with FP fusions: these are ultimately mutant alleles that may produce unexpected mutant phenotypes.

More recently, we described an FP fusion to the opposite N-terminal side, EGFP-US9. Viruses expressing this fusion are capable of anterograde axonal transport and spread in neurons, this fusion allows us to visualize the co-transport of EGFP-US9 and capsids in axons [79], and it has proven especially useful for immunoaffinity purification and proteomic identification of cellular factors involved in viral transport [37].

### 4.2. HSV-1 and PRV Capsid Protein VP26

VP26, the small capsid protein of HSV-1 and PRV, was one of the first herpesvirus proteins to be fused to a FP [41,46]. Since then, these capsid-tagged virus mutants have become a valuable tool to study virus particle transport in living cells. While VP26 is not essential in HSV-1 or PRV, there are differing reports on the effect of FP fusions to VP26. Some studies did not find any effect on virus replication kinetics [41,56], another reported moderate effects on cell-to-cell spread and pathogenesis *in vivo* [44], and yet another reported severe replication defects and reduction in neuro-invasiveness *in vivo,* comparable to a VP26-null virus [102]. A recent report has shown that some FP-VP26 fusions in HSV-1 severely attenuate virus replication, while others do not [45]. The reasons for these differences seem to be multifactorial, and here we will dissect what we believe are important factors that influence the viability of VP26 fusions.

#### 4.2.1. Compensatory Mutations that May Affect FP-VP26 Expression

It has been shown that the original HSV-1 strain containing an EGFP-VP26 fusion harbors an inadvertent 65 bp deletion upstream from the VP26 start codon [45]. Nagel *et al.* took the effort to recreate the virus mutant as originally designed [41] using a BAC recombination approach, and surprisingly found that this mutant was highly attenuated. Because the deleted region contains transcriptional enhancers [45], it is likely that this deletion is a compensatory mutation that causes reduced expression of the fusion protein.

**Figure 5:**
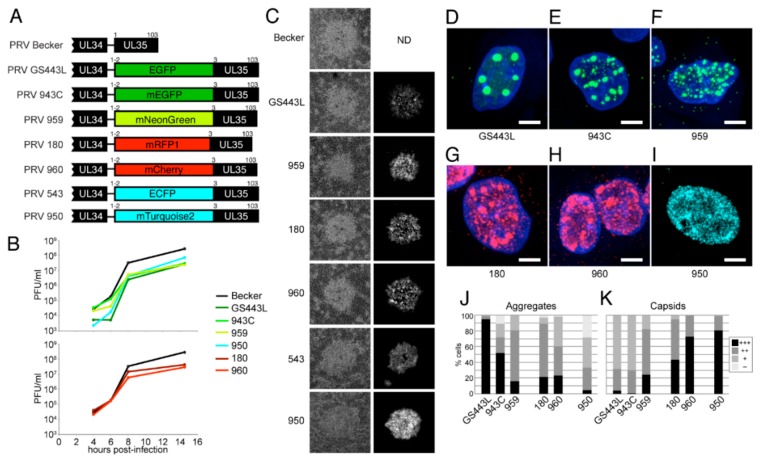
Fluorescent protein fusions to VP26, the small capsid protein, in pseudorabies virus (PRV). (**A**) Fluorescent protein coding sequences were inserted by homologous recombination between codons 2–3 of the PRV UL35 (VP26) gene. (**B**) Single-step virus replication. Parallel cell cultures were infected in triplicate with the indicated viruses, cells and supernatants were harvested at indicated times, and infectious virus titer was measured by plaque assay. Recombinants have ~1 log defect in end-point virus titer relative to parental PRV Becker. (**C**) Comparison of plaque size and fluorescence intensity. PK15 cells were infected with the indicated viruses, and one representative plaque is shown. Note that plaque sizes of recombinants are slightly smaller than that of parental PRV Becker. Newer FP variants are generally brighter than their predecessors: PRV 959 is brighter than PRV GS443L, and PRV 950 is brighter than PRV 543, but PRV 180 is similar to PRV 960. (**D**–**K**) Capsid assembly and nuclear aggregate formation. PtK2 cells (ATCC CCL-56) were infected with the indicated viruses, fixed at 6 h post-infection, and imaged by confocal microscopy. (**D**–**I**) Maximum intensity projection of representative nuclei containing individual fluorescent PRV capsids (smallest puncta) and large capsid protein aggregates (*i.e*., “assemblons”). Scale bars are 5 µm. (**J**–**K**) Quantification of intranuclear capsids and aggregates. More than 100 cells infected with each indicated virus were manually scored. (**J**) Intranuclear aggregates were scored as follows: +++, very large aggregates, as in panel D; ++, mid-sized aggregates, as in panels E-H; +, small aggregates, as in panel I; –, no aggregates. (**K**) Intranuclear capsid concentration was scored as follows: +++, dense, as in panels G-I; ++, many individual capsids, as in panel F; +, few individual capsids, as in panels D–E.

PRV seems to show a similar phenomenon. The original PRV EGFP-VP26 mutant (PRV GS443, [46]) was constructed in *Escherichia* *coli* by BAC recombination. Upon reconstitution of replicating virus, mixed plaque phenotypes were observed, with an approximately equal number of large and small plaques. Both phenotypes were stable during virus propagation; however, transfection of purified nucleocapsid DNA from these two populations yielded the surprising result that DNA from small plaque clones could again generate small and large plaques, while DNA from large plaque clones (PRV GS443L) only produced large plaques. Subsequent restriction analysis of nucleocapsid DNA revealed a SalI restriction fragment length polymorphism near the UL35 (VP26) gene [103]. We independently constructed VP26 fusions to mEGFP and mCherry (see Figure 5), and found similar results, suggesting that some adaptive mutation near the FP-VP26 coding region is needed to compensate for the defects caused by FP fusion. We sequenced the genome of several recombinants expressing FP-VP26 using next-generation sequencing methods, but were unable to clearly identify a putative compensatory mutation [150]. It is likely that the putative compensatory mutation is associated with expansion or contraction of a short sequence repeat region downstream of the UL35 (VP26) gene. A previous study identified copy number variation between common laboratory strains of PRV within this short sequence repeat region, and the repeated sequence contains a predicted binding site for the host transcriptional repressor CTCF [151]. Reconstitution of replicating virus by transfection of BAC or nucleocapsid DNA might increase the frequency of recombination in this repeat region, which may modulate the expression of the FP-VP26 fusion. It is possible that such variation explains the apparent differences in replication kinetics reported by different laboratories [44,56,102]. Further investigation into these putative compensatory mutations, and, in particular, development of new sequencing methods that can better cope with highly repetitive and GC-rich alpha herpesvirus genomes are needed.

#### 4.2.2. Fluorescent Protein Dimerization

Nagel *et al.* also found another piece of the puzzle. While the EGFP-VP26 HSV-1 mutant lacking the 65 bp upstream deletion was highly attenuated, another mutant expressing ECFP on the same genetic background only showed slight attenuation, even though ECFP and EGFP differ by only a few amino acids. Why did the ECFP recombinant grow while the EGFP recombinant could not? The solution seems to be that ECFP has a 30-fold higher dissociation constant (K_d_ ~3 mM) than EGFP (K_d_ ~0.1 mM), meaning that ECFP only dimerizes at a 30-fold higher concentration. This difference in dimerization affinity is a result of the N146I mutation present in ECFP, and newer cyan variants like mTurquoise2 contain a similar N146F mutation. Consistent with this observation, another HSV-1 recombinant expressing VP26 fused to mVenus also replicated well despite having a wild-type 65 bp upstream promoter region [45,82]. mVenus, like most newer *Aequorea* derivatives, contains the A206K mutation that further increases the dissociation constant to 74 mM [6]. Together, both of these factors, compensatory mutations reducing FP-VP26 expression and FP variants with reduced dimerization affinity, appear to improve replication of virus recombinants expressing FP-VP26 fusions.

#### 4.2.3. Assemblons

Both of these factors also affect intranuclear aggregation of capsid proteins. Nuclear aggregates formed by capsid proteins were found in cells infected with wild-type alpha herpesviruses, even before the first VP26 fusion was created. At that time it was believed that these were sites of capsid assembly, and these structures were dubbed “assemblons” [152]. However, Nagel *et al*. showed that “assemblons” are not sites of capsid assembly, but rather dead-end aggregations of capsid proteins, an idea also supported by earlier reports [153,154]. The early EGFP fusions to VP26 in HSV-1 show an especially strong disposition to form large “assemblons” earlier in infection [41,155], and the size and number of these aggregates correlates with FP dimerization affinity, with EGFP fusions making more aggregates than ECFP, mVenus, or mRFP1 fusions [45]. It therefore seems that FP-VP26 expression level and FP dimerization propensity are two sides of the same coin: A more strongly dimerizing FP forms aggregates even at lower expression levels, while a more monomeric FP forms fewer aggregates at higher expression levels. In mouse cytomegalovirus, an inducibly and ectopically expressed EGFP fusion to SCP (small capsid protein, the beta herpesvirus ortholog of VP26) acts as a very potent dominant negative [156], suggesting that the EGFP-SCP fusion can bind and sequester wild-type capsid proteins, inhibiting capsid assembly. It is possible that FP-VP26 fusions in the alpha herpesviruses also act as dominant negatives in a less strict sense, by sequestering capsid proteins into nuclear aggregates. This idea was suggested by Nagel *et al.*, and is supported by their identification of five different capsid proteins in the intranuclear aggregates. This would explain why EGFP-VP26 fusions, which form severe intranuclear aggregates also assemble relatively few individual intranuclear capsids [45] (Figure 5J–K).

The question remains, why are VP26 fusions so prone to induce protein aggregations? Capsid formation can be described as an irreversible phase separation. Typically, biological systems take advantage of phase separations by balancing at the “tipping point”, allowing rapid responses to small concentration changes. Capsid formation might be similar such that capsids rapidly assemble as soon as a critical capsid protein concentration is reached. Adding a dimerizing FP to capsid proteins might tip the balance, resulting in premature aggregation of capsid proteins.

#### 4.2.4. Description and Characterization of New VP26 Fusions in PRV

We generated a palette of different FP-VP26 fusions in PRV to identify those most suitable for live cell imaging (Figure 5A). FP coding sequences were inserted by homologous recombination between codons 2–3 of the PRV UL35 (VP26) gene, as previously described [64]. PRV GS443L ([46], and see above), PRV 959 [157], PRV 180 [64], PRV 543 [158], and PRV 950 [36] were previously described. Newer FP variants were chosen based on the criteria discussed in Section 3 (e.g., mEGFP, mNeonGreen, mCherry, and mTurquoise2), and were compared to older variants (e.g., EGFP, mRFP1, and ECFP). We found that all VP26 fusions caused an ~10-fold defect in single-step virus replication (Figure 5B), and slightly reduced plaque sizes (Figure 5C). Using identical imaging parameters (within each spectral class), we found that newer FP variants are generally brighter than their predecessors (e.g., PRV 959 is brighter than PRV GS443L, and PRV 950 is brighter than PRV 543; however, PRV 180 is similar to PRV 960) (Figure 5C).

We next imaged cells by spinning disk confocal microscopy at 6 h post-infection to determine the extent of capsid assembly and aggregate formation. As described above, and in agreement with HSV-1 VP26 fusions [45], monomeric FPs produce generally less aggregation and more individual intranuclear capsids (Figure 5D–K). By these criteria, the mTurquoise2 fusion appears to perform the best, and mRFP1 and mCherry fusions perform similarly well (Figure 5G–K). mEGFP, containing the monomerizing A206K mutation, appears to be a modest improvement over EGFP (Figure 5 D–E,J–K); however, mNeonGreen, which not derived from *Aequorea,* appears to be the best green capsid tag to date (Figure 5F,J–K). In particular, the combination of brightness, photostability, efficient excitation by powerful and inexpensive 470 nm LEDs makes the mNeonGreen-VP26 fusion especially suited for tracking capsid transport in neurons [157].

## 5. Comparison to Immunofluorescence

First described in 1942, the method of detecting and localizing antigens in cells or tissues using fluorescent antibodies has proven invaluable in molecular and cell biology [159]. Immunofluorescence was first used to visualize VZV infected tissues and cells in 1954 [160], and HSV in 1956 [161]. However, to visualize intracellular antigens, immunofluorescence protocols require destructive fixation and permeabilization steps, typically accomplished by aldehyde crosslinking of proteins, protein precipitation and lipid extraction by organic solvents, and/or lipid extraction using detergents. Variability in fixation and permeabilization can easily introduce artifacts, ranging from over-extraction leading to loss or relocalization of proteins of interest, to under-permeabilization leading to poor antibody accessibility [162].

Due to their complex architecture, including lipid envelope and thick tegument layer, immunofluorescence artifacts may be particularly problematic when visualizing assembled alpha herpesvirus particles. In one study, HSV-1 particles incorporating an mRFP1-VP26 capsid tag were adhered to a coverslip, fixed, permeabilized, and immunostained to detect the major capsid protein VP5. Naked capsids purified from the nuclei of infected cells were readily stained by anti-VP5 antibodies; however, fewer than half of assembled virions released from infected cells and immunostained *in situ* were detected by immunofluorescence [163]. Importantly, immunofluorescence detection was restored when assembled virions were purified or concentrated by ultracentrifugation [163,164]. Others have observed a time-dependent structural reorganization that alters the sensitivity of the virion tegument to detergent extraction [165]. In light of these observations, it seems likely that age and mechanical forces on virus particles can affect their permeability during immunostaining procedures. This inefficiency of immunofluorescence is likely compounded inside infected cells, where additional membrane layers and cellular proteins may impede permeability. Indeed, inefficient immunodetection of HSV-1 virions inside infected cells has been reported [166].

There is currently a debate in the alpha herpesvirus literature whether nascent virus particles are fully assembled prior to long distance anterograde transport in axons (the “married model”), or if capsids and membranes are transported separately before envelopment at distal sites (the “separate model”). The details of this debate are discussed elsewhere [167,168,169], but if immunostaining of fully-assembled virus particles inside axons is inefficient, this could lead to an underestimation of fully-assembled virions transporting in axons.

It is clear that immunofluorescence and FP-based approaches have different strengths and weaknesses. Immunofluorescence approaches suffer from variability in immunostaining, but have the advantage of detecting native protein at normal expression levels. Plus there already exists a wealth of well-characterized antibodies targeting viral and cellular proteins. However, while FP fusions may produce unexpected mutant phenotypes, they do enable a dynamic view in live cells, and enable a wide range of specialized techniques to better dissect complex multistep viral processes.

## 6. Beyond Traditional Fluorescence Microscopy

### 6.1. Fluorescent Protein Fusions as Proteomics Probes

Most viral and cellular biological processes are mediated by protein-protein interactions and protein complexes. Immunoaffinity purification coupled to mass spectrometry provides a powerful approach to isolate and characterize protein complexes and identify protein interaction networks. Although immunoaffinity purification can be performed using antibodies raised against viral proteins themselves, the alpha herpesviruses express a multitude of viral proteins, and antibodies produced using standard methods may not be of sufficient specificity and affinity for effective immunoisolation. In addition, antibodies against specific proteins may interfere with protein-protein interactions of interest. Alternatively, genetically fusing epitope tags to viral proteins can facilitate immunoaffinity purification of a variety of viral proteins using a single well-characterized high-affinity antibody. Peptide epitope tags are highly effective [170]; however, FPs have the distinct advantage of serving as both an epitope tag for immunoaffinity purification and a fluorescent probe for live-cell fluorescence microscopy. Combining these methods allows the identification of protein-protein interactions and provides a richer understanding of the spatiotemporal dynamics of these protein-protein interactions [171,172]. As described above, this approach allowed our laboratory to identify interaction between PRV US9 and the cellular kinesin-3 motor KIF1A, and connect this motor recruitment event to the dynamic axonal transport of virus particles [37].

### 6.2. Flow Virometry

Cell sorting was established over 50 years ago [173], modified to allow for fluorescence–assisted sorting (FACS) [174], and is now used in a wide array of biomedical research. Over the years, size restrictions were improved to reliably sort bacterial cells [175] and subcellular eukaryotic organelles (fluorescence-assisted organelle sorting, FAOS), including mitochondria, lysosomes, endosomes, and exosomes [176,177,178,179]. Importantly, FAOS operates in the size range of larger virions (>100 nm) and can be adapted for virus research. Pioneering work in the field of “flow virometry” has greatly improved the throughput and sensitivity of large-scale virus detection and discovery. Modern FACS equipment, improved sheath water filtration, and more sensitive optics now allow the detection of viral particles less than 60 nm [180]. Detection of individual virus particles by FACS can principally be done through two methods: One method uses the light scattering properties of the particle. Early reports indicated that viruses with distinct morphologies (e.g., T2 phage) can be identified solely based on their light scattering [181]. The other method uses fluorescent dyes, or a combination of light scattering and fluorescent dyes. Fluorescent labeling has been used to reliably detect and quantify viruses like HSV, CMV, adenovirus, influenza A, RSV, rubella, coronavirus, dengue, and parainfluenza [182,183]. In many cases, non-specific nucleic acid and protein-binding fluorescent dyes are used to label virus particles, but some reports also describe the use of specific antibodies to detect HIV-1, poliovirus, dengue virus [184,185,186]. These fluorescent labeling methods may affect particle infectivity, which is of minor concern if virometry is used only to detect and measure virus particles. However, if virus particles need to be sorted retaining infectivity, for example, to measure infectivity of different subpopulations, FP fusions might be a more suitable choice. As a proof-of-principle, the Lippé laboratory analyzed HSV-1 capsids containing EGFP-VP26 [187], and more recently sorted infectious HSV-1 virions containing EGFP-tagged tegument proteins [188]. Just as FACS analysis provides a population distribution of cellular properties rather than a bulk average, “flow virometry” will allow the alpha herpesvirus field to better understand the variability within subpopulations of virus particles.

### 6.3. Herpes Past the Diffraction Barrier

For over a century, light microscopy resolution was limited by the Abbe diffraction barrier [189], which means in practice that structures closer than ~200 nm cannot be resolved. However, new super-resolution fluorescence microscopy methods have been developed, pushing these limits to under 20 nm. This allows more precise localization and discrimination of delicate cellular structures, providing greater insights into biological processes (reviewed in [190]). Virologists have now begun to exploit the potential of super-resolution microscopy, the progress of which is reviewed in detail elsewhere [191,192,193]. While some popular super-resolution techniques like stochastic optical reconstruction microscopy (STORM) and stimulated emission depletion (STED) rely on chemical fluorophores, other methods, like photoactivated localization microscopy (PALM) and structured illumination microscopy (SIM) are compatible with FPs. PALM does not require chemical fixation, permeabilization, and immunostaining, so samples can be analyzed without many of the caveats described above (see Section 5). This method might, for example, be useful to understand the dense network of proteins in the herpesvirus tegument, or virus-related cellular structures like “assemblons”. The first steps have been taken in this direction, measuring the asymmetric distribution of viral structural proteins in PRV particles using a related method [50], and radial distribution of tegument proteins in HSV-1 particles using STORM [194].

Most super-resolution techniques, however, are limited by very slow acquisition rates. SIM is presently the only method that can deliver super-resolution data at high enough frame rates to follow virus dynamics in infected cells. However, in our experience, most FP-tagged virus mutants were incompatible with this method due to rapid photobleaching [195]. Further developments are needed to optimize FP brightness and photostability, and optimize the speed and sensitivity of super-resolution techniques to allow a super-resolution view of the dynamic virus replication cycle.
